# Endothelial Mechanotransduction, Redox Signaling and the Regulation of Vascular Inflammatory Pathways

**DOI:** 10.3389/fphys.2018.00524

**Published:** 2018-06-07

**Authors:** Shampa Chatterjee

**Affiliations:** Department of Physiology, Perelman School of Medicine, Institute for Environmental Medicine, University of Pennsylvania, Philadelphia, PA, United States

**Keywords:** endothelial mechanotransduction, inflammation, redox signals, revascularization, vascular disease

## Abstract

The endothelium that lines the interior of blood vessels is directly exposed to blood flow. The shear stress arising from blood flow is “sensed” by the endothelium and is “transduced” into biochemical signals that eventually control vascular tone and homeostasis. Sensing and transduction of physical forces occur via signaling processes whereby the forces associated with blood flow are “*sensed”* by a mechanotransduction machinery comprising of several endothelial cell elements. Endothelial “*sensing*” involves converting the physical cues into cellular signaling events such as altered membrane potential and activation of kinases, which are “*transmission”* signals that cause oxidant production. Oxidants produced are the “transducers” of the mechanical signals? What is the function of these oxidants/redox signals? Extensive data from various studies indicate that redox signals initiate inflammation signaling pathways which in turn can compromise vascular health. Thus, inflammation, a major response to infection or endotoxins, can also be initiated by the endothelium in response to various flow patterns ranging from aberrant flow to alteration of flow such as cessation or sudden increase in blood flow. Indeed, our work has shown that endothelial mechanotransduction signaling pathways participate in generation of redox signals that affect the oxidant and inflammation status of cells. Our goal in this review article is to summarize the endothelial mechanotransduction pathways that are activated with stop of blood flow and with aberrant flow patterns; in doing so we focus on the complex link between mechanical forces and inflammation on the endothelium. Since this “inflammation susceptible” phenotype is emerging as a trigger for pathologies ranging from atherosclerosis to rejection post-organ transplant, an understanding of the endothelial machinery that triggers these processes is very crucial and timely.

## Introduction

The vascular endothelium serves as an interface between the blood and the tissue. This layer of cells is increasingly recognized as a critical component of vascular health. The endothelial regulation of vasodilation and contraction, of vascular permeability and of inflammation and immune signaling is critical to vascular health which in turn is pivotal to our survival.

**Figure 1:**
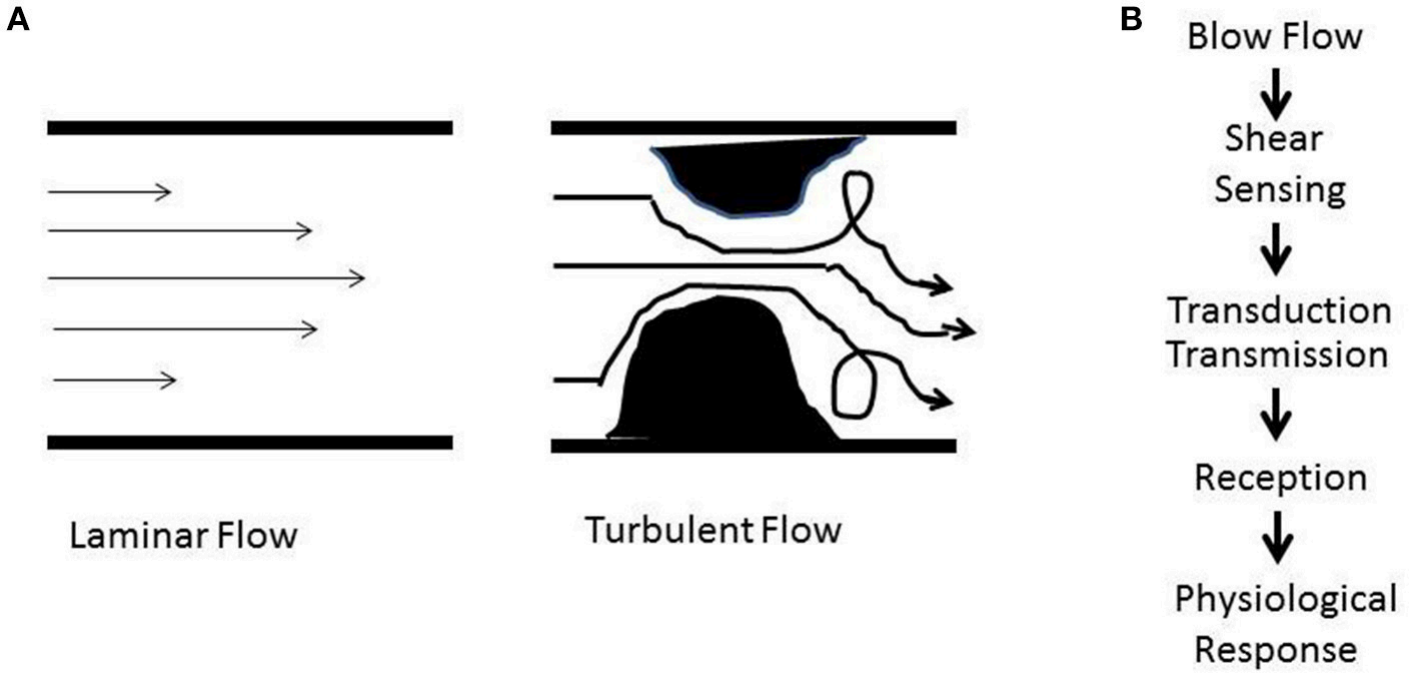
**(A)** Schematic showing patterns of blood flow. The laminar or regular streamlined blood flow is high in the center of the vessel and faces drag or friction along the walls. Obstructive or stasis causes turbulent flow that is characterized by eddies and whirlpools. **(B)** Shear stress induced signaling involves sensing (mechanotransduction), transduction and transmission (ROS, NO), reception (cellular receptors), and physiological response (atherosclerosis, proliferation, angiogenesis, inflammation).

Because the endothelial layer lines blood vessels, it can “interact” with the forces arising from the flow of blood (Davies, 2009; Sandoo et al., 2010). Blood flow is associated with tangential shear stress and circumferential wall stretch. In major vessels such as aorta or arteries that are made up of the endothelial lining (intima) and the smooth muscle layer (media and adventitia), the frictional force per unit area from flowing blood, acts on the endothelial cells (ECs) and gives rise to shear stress while the blood pressure exerts a circumferential stretch normal to the vessel wall (Davies, 1993, 1995; Davies et al., 1997). However, microvessels consisting of an endothelial layer only, experience only shear stress. Blood flow in the form of both shear stress and stretch act on the endothelial layer and vessel wall respectively and regulate a number of pathways that participate in maintenance of vascular tone and function. Studies on the effects of shear stress associated with blood flow point to its role on regulating a number of physiological responses (Dewey et al., 1981; Barakat, 1999; Chiu et al., 2007). *In vitro* and *in vivo* studies have shown that these responses range from organization of the vascular tree during the various stages of embryogenesis and development of the fetal circulatory system to adult vascular tone and function (Tardy et al., 1997; Morgan et al., 1998; Topper and Gimbrone, 1999; Matharu et al., 2008). However, disruption of this laminar or regular shear stress (Figure 1B) is also a driving factor for the development of several pathologies. These occur when aberrant shear or stop of shear leads to activation of signaling cascades that cause vascular dysfunction, inflammation, and injury.

How does shear stress affect vascular development and function? The answer lies in the endothelium, the layer of cells that are in direct contact with blood flow. Work over the past two decades has demonstrated that the endothelium is equipped with a “sensing” machinery that can “sense” the force associated with blood flow and integrate it across the vasculature in the form of biochemical signals (Chatterjee and Fisher, 2014a,b,b). To date, our knowledge of the “sensing” machinery or mechanosensors is incomplete. Work from several groups including our own (Chien, 2007; Noel et al., 2013) indicate the involvement of several possible candidates that may work separately on in concert. These potential mechanosensors are cell surface and/or cytoplasmic receptors, ion channels, kinases, integrins, and extracellular matrix components (Rizzo et al., 2003; Weinbaum et al., 2003; Ingber, 2006; Wang et al., 2009). Additionally there are multimeric complexes with several mechanosensing moieties that act together to sense alterations in flow (Tzima et al., 2005; Noel et al., 2013). The eventual effect of mechanosensing is a long term adaptive response. In regions of laminar or streamlined blood flow, it maintains vascular tone while in regions where the flow is erratic and turbulent (as shown in Figure 1A) it causes onset of inflammation signaling. We found that when blood flow ceases, such as with the presence of a clot, the sudden cessation of shear drives cell proliferation and angiogenesis (Browning et al., 2014).

In this review, we discuss the flow driven mechanosensing pathway and its role in induction and amplification of vascular signaling. In doing so, we focus on the elements of “sensing,” and “transduction” that lead to activation of signaling pathways that alter endothelial phenotype (Browning et al., 2014; Chatterjee and Fisher, 2014b; Tao et al., 2016). These pathways that cause production of cellular adhesion molecules (CAMs), pro-inflammatory cytokines as well as activation of pro-inflammatory and angiogenic transcription factors eventually drive endothelial inflammation, vascularization, and remodeling (Browning et al., 2014; Dorland and Huveneers, 2017). Understanding this complex link between mechanical forces of blood flow and inflammation on the endothelium, informs us on the development of pathologies ranging from atherosclerosis to rejection post-organ transplant.

## The vascular endothelium as a “sensor” of blood flow (sensing, transduction, transmission, reception, response)

The vascular system, a conduit and connector of blood flow across organs, is a network that integrates biochemical and biophysical signals via systemic transport of blood, nutrients and inflammatory, pathogenic moieties across the body. Thus the endothelium that lines the vascular wall serves as an extensive interface where chemical and mechanical stimuli in blood interact directly with a cellular layer and “convey” signals into tissues. In addition, the endothelium is recognized as an initiator and converging site of inflammation, a pivotal event in organ injury. In an adult human, the surface area of the entire endothelium is 3,000 m^2^ which is equivalent to at least six tennis courts (van Hinsbergh, 2012; Yau et al., 2015). Comprising of one trillion endothelial cells, which weigh more than 100 g, the endothelium can be considered to an extensive and dynamic organ that pervades the entire body. Besides regulating the vasomotor tone, the endothelium controls cellular and nutrient trafficking from the blood into the tissue, maintains homeostasis (i.e., a balance between pro- and anti-coagulant activity of blood and pro-and anti-inflammatory environment in tissues.). To enable its functions, the endothelium must be able to receive “cues” from the local environment in the form of chemotransduction and mechanotransduction. Of note, chemotransduction has been reviewed by us elsewhere (Browning et al., 2012).

At a conceptual level, it is clear that mechanotransduction would involve sensing by the structural components of the cell, its transduction into a biochemical signal, and the effects of this signal on the endothelium and surrounding environment. This is summarized in Figure 1B (Chatterjee and Fisher, 2014b) as (a) the **force** of blood flow (b) **sensing** of the force via cellular structures; (c) **transduction** of the force via signaling molecules; (d) **propagation** of the signal and its **reception** by “cellular receptors”; and (e) an eventual physiological **response**.

## Signaling with flow and altered flow (sensing and transduction)

**Figure 2:**
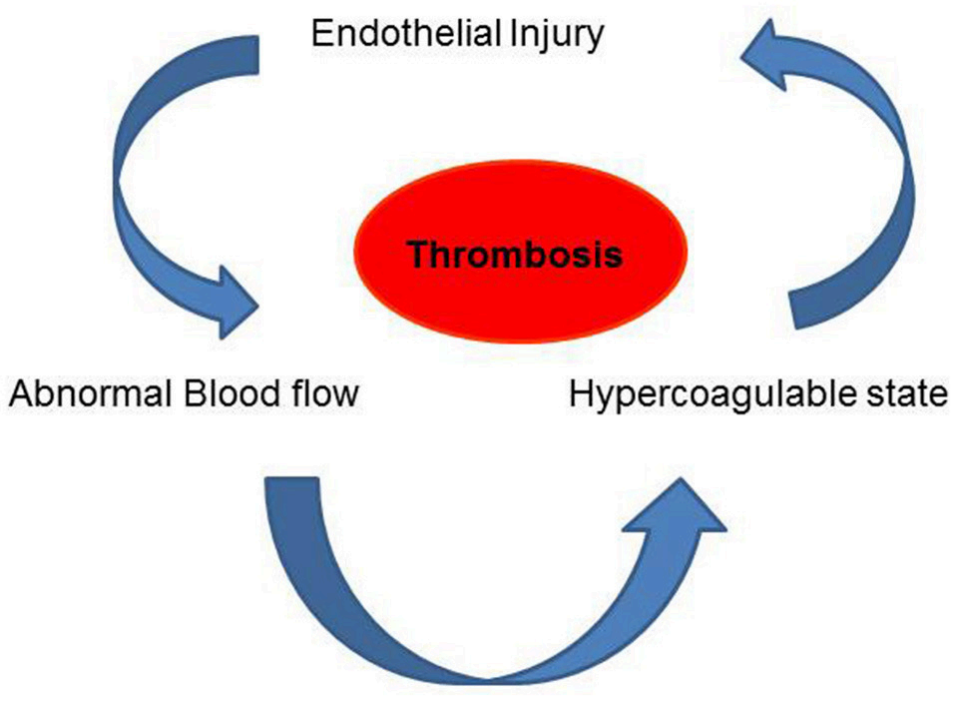
Virchow's Triad comprising of blood flow, vascular endothelial injury and the viscosity (constitution) of the blood are the three factors that are thought to contribute to thrombosis.

The pivotal importance of blood flow in maintenance of vascular health was well recognized as early as the Nineteenth century. Indeed, Virchow's triad had as its central tenet, three major players that were thought to contribute to thrombosis. These were alteration in blood flow, vascular endothelial injury, and alterations in the constitution of the blood (Figure 2; Lowe, 2003). We now know that all these are linked. The normal endothelium under uniform laminar shear stress produces nitric oxide (NO), prostacyclin (PGI2) and tissue plasminogen activator (t-PA) etc., all of which regulate vasoreactivity by controlling the adhesion of platelets, neutrophils etc. (Bagot and Arya, 2008; Aoki et al., 2016). This quiescent endothelial phenotype has been found to change under aberrant flow conditions (Hwang et al., 2003; Johnson et al., 2011). Alterations in shear associated with irregular blood flow such a low shear, eddies, recirculation of flow or by loss of shear can lead to signals that facilitate an activated endothelial phenotype (Hwang et al., 2003) which drives endothelial damage or injury by favoring adhesion of immune cells as well as by infiltration of plasma components such as cholesterol, low density lipoprotein (LDL), and fibrinogen. All these events correlate to increase in blood viscosity. These collective changes can by promoting an atherogenic phenotype eventually lead to thrombosis (Davies, 1995, 2008; Davies et al., 1995).

Thus, the hemodynamics of blood flow, the signaling associated with shear stress as well as the intersection of these signals with risk factors and the effects of underlying genetic control need to be studied for a better understanding of the **blood flow-mechanosignaling-remodeling-disease** paradigm.

At the center of this paradigm is endothelial dysfunction that arises from the loss of equilibrium between pro- and anti-oxidant signaling thereby producing oxidative stress. Oxidative stress or pro-oxidant load arises from redox signals such as reactive oxygen species (ROS) and reactive nitrogen species [RNS such as NO, peroxynitrite (ONOO−), nitrous oxide (^.^NO_2_), N_2_O_3_] as well as the activation of pro-inflammatory or pro-angiogenic pathways. Oxidative stress signals triggered by altered shear compromise endothelial structure thus driving endothelial dysfunction, the major etiology in diseases of the vasculature (Higashi et al., 2014). In the subsequent sections, we elaborate on the shear signaling initiated with various flow patterns and how these can promote atherosclerosis by either increasing pro-atherogenic or diminishing anti-atherogenic activities (Davies et al., 1995; Dai et al., 2004; Davies, 2008).

The pattern and magnitude of shear stress varies according to the vascular bed and the location of that bed in the vascular tree. In straight vessels, where the flow is laminar i.e., parallel to the vessel, the magnitude of shear stress is directly proportional to the viscosity of blood and inversely proportional to the third power of the (inner) radius of the blood vessel (Equations 1 and 2). Laminar flow maintains vessel function but it is altered flow in the form of increase, decrease or turbulent/disturbed (recirculating flow or eddies) that leads to signals that drive remodeling and disease.

Shear stress varies according to the vascular bed and species, with magnitude of shear being much higher in mouse vessels as compared to humans (Table 1). In arteries, capillaries and veins, the endothelium is exposed to various levels of shear stress ranging from 1 to 70 dyn/cm^2^. In arteries, shear ranges between 10 and 70 dynes/cm^2^ while in veins it is ~1–6 dynes/cm^2^. Overall in most arteries, shear stress is maintained between 10 and 20 dynes/cm^2^ (Natarajan et al., 2016).

(1) $$\begin{array}{l} \text{Shear stress in a vessel}\left(\tau \right)=4\mu \text{Q}/\pi {\text{r}}^{3}, \end{array}$$

where μ is the blood viscosity, Q the blood flow, π the ratio of the circumference of a circle to its diameter, and r the radius of the blood vessel.

(2) $$\begin{array}{l} \text{Shear Stress in parallel plate chamber}\left(\tau \right)=6\mu \text{Q}/{\text{bh}}^{2} \end{array}$$

where μ is the fluid viscosity, Q the blood flow, b is the width of the chamber and h is the distance between plates.

**Table 1:** Shear stress in select human blood vessels^*^.

**Vessel**	**Shear stress dyn/cm^2^**
Arteries	10–60
Veins	1–10
Stenosis in arteries	>100
High Stenosis in arteries	>1,000
Ascending Aorta	12
Descending Aorta	5–8
Pulmonary Artery	5
Small vein	11
Large vein	5

* *Approximate normal values from studies reported by Samet and Lelkes, 1999; Waite and Fine, 2007)*.

**Figure 3:**
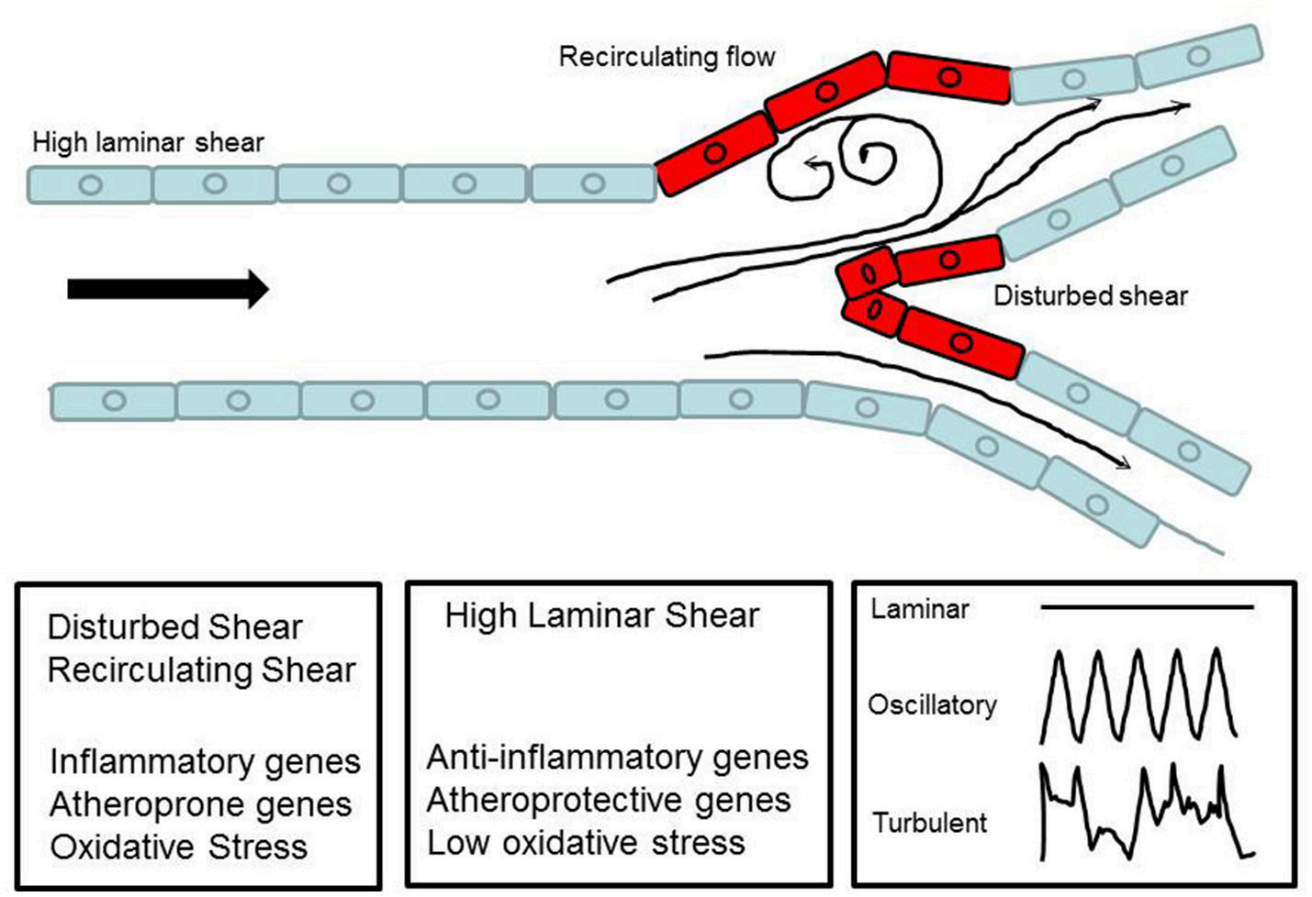
Schematic diagram to show laminar flow in a major vessel and disturbed flow at a bifurcation. The diagram illustrates how blood flow forms recirculatory patterns and eddies at the curvatures.

Non-laminar flow implies patterns of disturbed flow that occur at branch points, curvatures of large arteries, and the aortic arch and also with anastomosis/stent insertions as well as surgical manipulations associated with ischemia-reperfusion in procedures such as organ transplantation. These disturbances, in the form of oscillatory flow, retrograde flow and eddies, lead to non-uniform or irregular shear along the vessel wall. At branch points, disturbed flow occurs when high shear stress is impeded thus creating sites of abnormally low and high shear stress (Figure 3). This is sensed by the ECs. The resultant signaling causes the activation of a cascade that drives an inflammatory phenotype. This is in the form of a procoagulent surface, production of inflammatory cytokines and cell proliferation. Eventually cell proliferation (of both endothelial and smooth muscle cell) results in remodeling of the blood vessel (Tsao et al., 1996; Dirksen et al., 1998; Nerem et al., 1998; Gimbrone et al., 2000).

Mechanosignaling with shear stress has been well studied using various models of flow *in vitro* and *in situ* (Davies et al., 1995; Albuquerque et al., 2000; Tzima et al., 2005; Chatterjee et al., 2008; Davies, 2008; Rezvan et al., 2011; Chatterjee and Fisher, 2014b). In recent years, *in vivo* models have also been employed to assess the short and long term effects of stop of blood flow or disturbed blood flow (Rezvan et al., 2011; Browning et al., 2014). However, most *in vitro* studies have been largely limited to “onset of flow” on ECs in culture. Such models do not accurately represent the *in vivo* state where the ECs are under continuous flow or under turbulent flow. We and others have thus used models with ECs adapted to flow (Chatterjee et al., 2012; Chatterjee and Fisher, 2014b). Yet others have employed models where flow is “stepped up” to create turbulent shear (Kwei et al., 2004). Additionally *in situ* and *in vivo* models have also been employed to evaluate the effects of shear signaling on vascular remodeling and function. Each of these models provides information on shear signaling, but has limitations in that these are confined to representing a particular shear signal, cell type, and vascular bed. Therefore, the findings across all these models need to be integrated in order to inform our knowledge of mechanosignaling. Outlined below are some of the models that have been routinely used in mechanotransduction studies.

### Models of endothelial mechanotransduction (Figure 4)

#### The flow adapted endothelial cell (FAEC) as an *in vitro* model to study mechanotransduction

**Figure 4:**
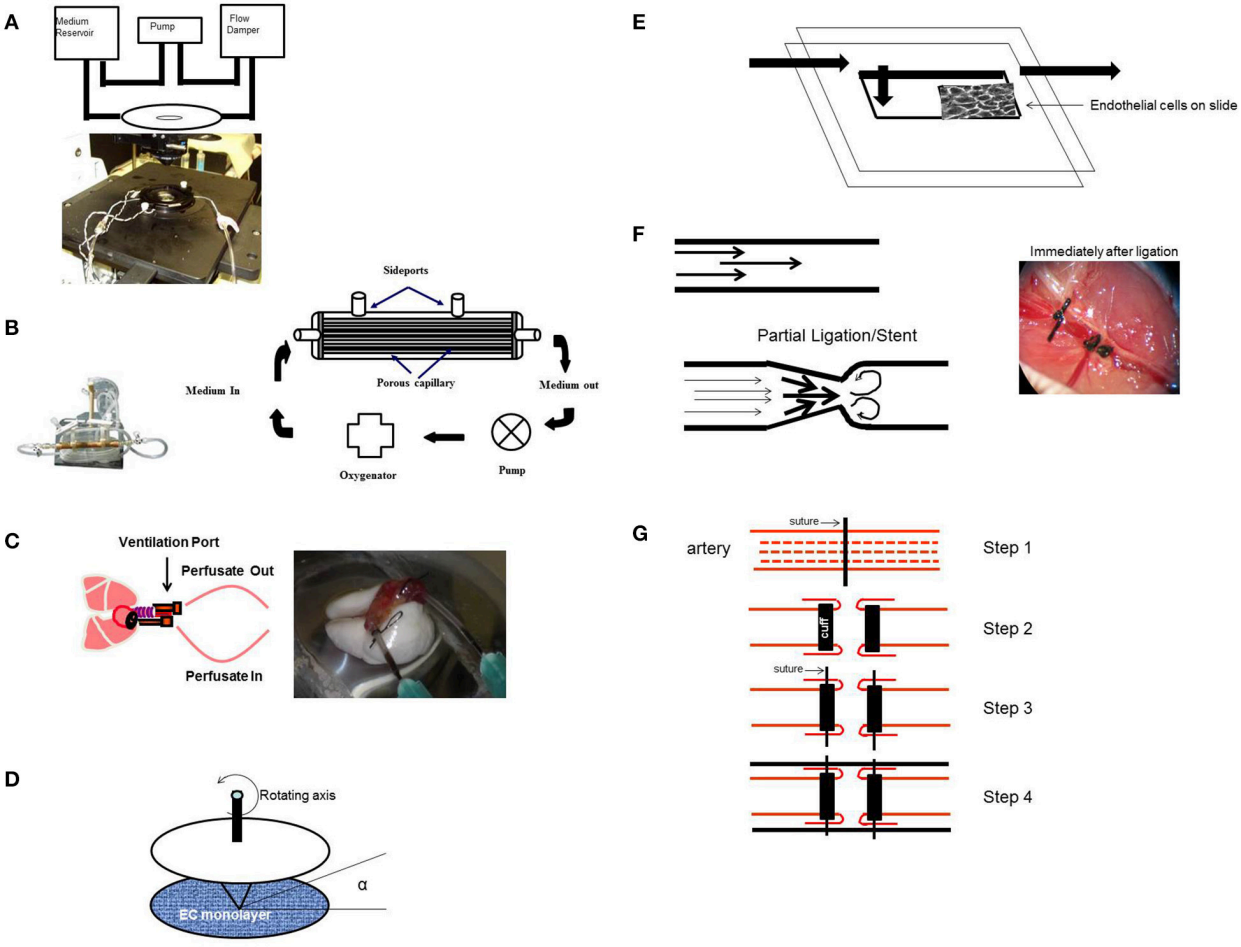
Experimental Models (*in vitro* and *in situ*) to study signaling with various patterns of flow. **(A)** *In vitro* flow system (parallel plate chamber) that allows for real time fluorescence microscopy with flow. Endothelial cells grown on coverslips are inserted into the chamber and kept under flow. After 24 h of flow adaptation, various signaling molecules can be monitored by the use of fluorescent dyes (that monitor membrane polarity or ROS production) and real time confocal microscopy. **(B)** Artificial capillary chamber consisting of narrow capillaries (polypropylene) to mimic blood vessels. Endothelial cells seeded into these capillaries and allowed to attach. During this attachment period, cells are fed via perfusion through abluminal ports. For flow adaptation, medium is perfused through luminal ports. **(C)** *In situ* (*ex vivo*) model of altered flow in the lung. Rat or mouse lungs cleared of blood are ventilated and perfused. The lungs are then preperfused with fluorescent dyes, placed on the stage of a microscope, and imaged for ROS, Ca^2+^, or NO, etc. **(D)** Cone-and-plate viscometer recapitulates the blood flow of the arterial system on endothelial cells. **(E)** *In vitro* model of disturbed flow whereby a step at the inlet in a parallel plate chamber can create eddies downstream of the step. **(F)** *In vivo* model of disturbed flow (partial ligation) or stop of flow (ligation) of arteries. Left: ligation or stents would lead to recirculation and eddies. Right: Complete ligation of femoral artery leads to stop of flow as would occur with a clot or thrombus. **(G)** Schema of a bypass graft. First (Step 1) the vessel is sutured and then dissected (Step 2) and cuffs are placed at the end, and segment of the vessel turned outwards to cover the cuff (Step 2). The cuff was kept in place with a suture (Step 3). Finally a vessel segment (vena cava vein) was grafted over the ends (Step 4). The larger diameter of the engrafted vein as compared to that of the smaller vessel, results in lowering of the shear stress in the engrafted vein. Lowered shear can facilitate an atherogenic phenotype [parts of this figure were originally published in Chatterjee et al. (2014), no permission required].

To monitor the signals activated by altered flow, we devised an *in vitro* flow adapted endothelial cell (FAEC) model whereby cells kept under flow for long periods (24–72 h) were studied (Figures 4A,B; Chatterjee et al., 2003, 2006; Tao et al., 2016). We reasoned that adapting ECs to flow for long periods mimics the *in vivo* endothelial bed and stop of flow would closely represent events associated with a blood clot or with organ transplantation (Tao et al., 2016). Our studies have thus evaluated mechanotransduction or mechanosignaling from the point of abrupt stop in shear. Using this *in vitro* stop of flow model, we reported that the earliest event with stop of flow was depolarization of the endothelial cell membrane that occurred due to the closure of a K_IR_ channel (of the 6 family) (Chatterjee et al., 2006, 2012).

#### The isolated perfused lung as an *in situ* model of mechanotransduction

Apart from the endothelial layer, the vascular bed is composed of smooth muscle cells and a basement matrix. Thus, mechanosensing events should be investigated in an intact vascular system. For this, our studies to date have used an isolated lung model; use of the intact lung provides a methodological advantage over other organs for “stop of flow” studies, since oxygenation can be maintained via ventilation of lungs (Figure 4C). In systemic organs, stop of blood flow would also result in hypoxia and thus mechanosignaling events would be overshadowed by hypoxia signaling. In the lung, stop of flow compromises the mechanical effects of blood flow alone as tissue pO2 values can be kept constant (pO2 ^*^140 mmHg) via ventilation. Indeed there was no decline in ATP levels with stop of blood flow in lungs (Al-Mehdi et al., 1997; Song et al., 2001).

#### Onset of flow models

Elsewhere, studies on mechanotransduction have used onset of flow models in the form of cone and plate device or parallel plate chambers (Figure 4D; Olesen et al., 1988; Franzoni et al., 2016); these studies have reported that the earliest event with onset of flow was endothelial cell membrane hyperpolarization due to the activation or opening of an inwardly rectifying K channel (K_IR_). Although the molecular identity of this channel is not clear, studies seem to indicate that it is a K_IR_2.1 channel (Hoger et al., 2002). Yet others have shown the activation of an outwardly rectifying chloride channel with onset of flow (Gautam et al., 2006).

#### Models of aberrant flow *in vitro* and *in vivo*

Disturbed flow often involves changes in direction of flow (retrograde flow) and *in vitro* models that reproduce these complex flow environments include (a) parallel plate flow chambers with a step at the inlet so as to create eddies downstream of the step (Figure 4E; Chiu and Chien, 2011) or with a variation in shear from the entrance (high shear) to the exit (zero shear) (Usami et al., 1993), (b) orbital shakers (Dardik et al., 2005), and (c) cone and plate viscometer. The rotating cone with a small angle creates shear on cells. Underneath the cone there would be an uniform shear field on the cultured cells, while outside the come (with higher centrifugal force) a disturbed flow pattern is created (Spruell and Baker, 2013).

*In vivo* models are

- Ligation and Partial Ligation Model (Rezvan et al., 2011):
  *In vivo* models of turbulent flow involve surgical manipulations in rodents in the form of complete or partial ligating vessels to redirect flow (Korshunov and Berk, 2003; Nam et al., 2009); indeed arteriovenous shunts have been routinely used (Asano et al., 2005). One such model is ligation of the left common carotid artery to increase laminar shear stress in the right carotid artery (Figure 4F, left; Alberts-Grill et al., 2012). Another model used by us to mimic stop of flow *in vivo*, is that of complete ligation of the femoral artery (Figure 4F, right; Browning et al., 2014).
- Venous Graft Occlusion Model:
  This involves generating an altered shear model in the form of a “high to low shear stress” model by performing an end to end transplantation of the external jugular vein to the common carotid artery (Zou et al., 1998; Figure 4G). Such a venous graft model leads to low shear and enhanced intimal thickening (Jiang et al., 2004) and when compared with the contralateral artery (control) is employed to study atherogenesis.

### Signaling with cessation of flow

**Figure 5:**
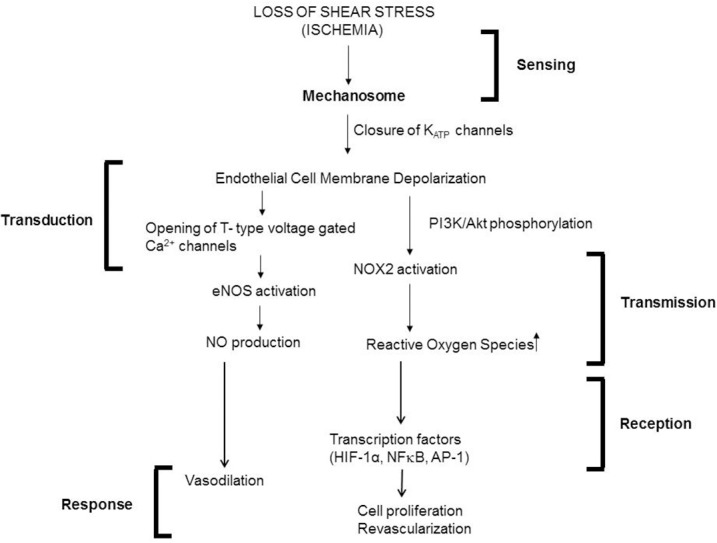
Mechanotransduction cascade in pulmonary vascular endothelium with stop of flow following the framework outlined in Figure 1. Mechanotransduction by endothelial cells occurs via the mechanosome complex comprising of caveolae-PECAM, VEGFR2 and VE-cadherin resulting in deactivation of KATP channel. This alteration in membrane potential results in activation of NOX2 and eNOS with consequent production of ROS and NO. These mediators result in vasodilation and revascularization probably as a signal to restore the stopped flow (originally published in Chatterjee and Fisher, 2014b, no permission required).

Our group has studied endothelial mechanosignaling from the point of cessation of flow (Chatterjee et al., 2006; Noel et al., 2013). Using both FAECs (adapted to flow at 1–5 dyn/cm^2^ characteristic of shear in the microvasculature) and isolated lungs, we observed that stop of flow leads to the onset of a signaling cascade, the earliest event of which is the depolarization of the endothelial cell membrane. We observed depolarization by the use of membrane polarity sensitive dyes as well as by measuring membrane currents (patch clamp) (Chatterjee et al., 2006). We discovered that depolarization occurred via a K_ATP_ channel (composed of an inwardly rectifying K^+^ channel of 6 family) that closed in response abrupt cessation of flow. The closure-depolarization event led to activation of a PI3Kinase-Akt pathway that caused assembly of the subunits of the enzyme NADPH oxidase 2 (NOX2) and subsequent production of ROS (Chatterjee et al., 2012). We also observed that depolarization of the endothelial membrane altered its potential from −80 to −60 mV to about −30 to −20 mV, at which potential there was activation of voltage gated calcium channels (VGCC). VGCC facilitates entry of calcium ions into the ECs leading to activation of endothelial nitric oxide synthase (eNOS) and subsequent NO production (Al-Mehdi et al., 2000; Figure 5).

Meanwhile, reaction of ROS with NO (generated by both stop and onset of shear) lead to generation of ONOO^−^ (Pacher et al., 2007; Golub and Pittman, 2014) which can alter endothelial phenotype by disrupting cellular signaling, as well as by inducing the activation of inflammatory pathways, such as NFκB and the mitogen activated protein kinase (MAPK) p38 MAPK. We showed that stop of shear led to activation of transcription factors NFκB and activator protein 1 (AP-1) (Wei et al., 1999, 2001).

### Signaling with onset of flow

Studies on endothelial mechanotransduction from the point of “onset of flow” also showed that the earliest event with start of flow was altered endothelial membrane potential in the form of hyperpolarization. This reportedly occurred via the opening of inwardly rectifying K channels of the 2.1 family (Olesen et al., 1988; Hoger et al., 2002). In other reports, the labs of Martin Schwartz and Eleni Tzima showed that exposing ECs to flow led to the activation of a mechanosensory complex comprising of the platelet endothelial cell adhesion molecule-1/vascular endothelial cadherin/vascular endothelial growth factor receptor 2 (Tzima et al., 2005). This led to conformational activation of integrins which (outside-in integrin activation) activates the transcription factor NFκB via focal adhesion kinase (FAK) dependent phosphorylation. Other reports have shown that similar to stop of flow, ROS is also produced with onset of flow. ROS is well established to activate NFκB via degradation of IκB (Gloire et al., 2006). In general, activation of NFκB facilitates inflammatory activation of the endothelium. With onset of flow both ROS and FAK signaling contributes to NFκB activation (Tzima et al., 2002).

However long term exposure of ECs to laminar flow eventually inhibits the NFκB as well as MAPK pathways. Shear activates the MAPK7 pathway which downregulates inflammatory entities through induction of the anti-inflammatory transcription factors, Kruppel-like factor 2 (KLF2) (Parmar et al., 2006) or KLF4 (Ohnesorge et al., 2010).

### Signaling with turbulent/aberrant flow

Turbulent and disturbed flow occurs in the form of recirculation eddies as well as retrograde or reverse flow at branchings of vessels and at inner curvatures of the aortic arch. In these regions, the endothelium experiences a different magnitude and direction in the flow pattern. Here too endothelial mechanosignaling via shear sensing machinery leads to “activation” of the ECs in the form of generation of redox signals (such as ROS and NO) and activation of pro-inflammatory transcription factors (Davies, 2009). Indeed Passerini et al. (2003) showed that in ECs adapted to flow, reverse flow of low magnitude elevated the expression of inflammatory moieties such as transforming growth factor–beta (TGF-β) and platelet derived growth factor PDGF-B but reduced the production of NO. In porcine arteries, both NO and ROS were found to be produced as a directional response to shear stress; with superoxide production decreasing NO availability during reversed flow (Godbole et al., 2009).

Pro-inflammatory transcription factors such as NFκB and AP-1 lead to high expression of inflammation moieties such as CAMs, monocyte chemotactic protein-1 or MCP-1 (that facilitates monocyte recruitment, adherence, and infiltration into the endothelial layer) (Hsiai et al., 2001, 2003) and growth factors such as vascular endothelial growth factor (VEGF) as well as platelet derived growth factors (PDGF) that increase endothelial proliferation and migration. Studies from the Davies group have shown that disturbed and laminar flow patterns result in different responses on ECs (Davies et al., 1997; Davies, 2008). This leads to preferential localization of atherosclerotic lesions at arterial branches and curvatures while the straight parts of the arterial tree are spared (Davies et al., 2010). However, it needs to be noted that development of atherosclerotic lesions depends of the prevalence of other risk factors in addition to the patterns of blood flow. For instance, in normal adult pigs, regions of turbulent flow show the expression of both atherosclerosis-susceptible (proinflammatory, procoagulant) and atherosclerosis-protective (antioxidant and anticoagulative) genes (Davies, 2009). Thus although disturbed flow characteristics can predict the possibility of atherosclerosis, the presence of protective genes as well as the absence of risk factors may keep this in check.

Overall, signaling with shear (laminar, turbulent, cessation of shear) involves the activation of several moieties including NFκB, AP-1, MAPK, TGF-β pathways etc. Their (often) paradoxical roles in inflammation, endothelial dysfunction and protection arise due to orchestration and cross talk between various pathways and their intersection with redox signaling molecules on the endothelium. These determine the eventual long term response in the form of inflammation, remodeling, atherosclerosis or maintenance of vascular tone, and vasodilatation.

### The mechanosensing machinery in cells and tissues

#### Tensegrity

The cell as a tensegrity structure: According to this theory the cell with its membrane and cytosolic components acts as a single integrated structure which senses physical forces through tensegrity (tensional-integrity). The load bearing cytoskeletal elements conveys the mechanical stress into the cytoplasm and nucleus (Ingber, 2006). The major cytoskeletal elements in the cell are the actin-myosin machinery which forms filaments, microfilaments microtubules and intermediate filaments. Studies have shown that the cellular response to mechanical stress applied to the cell surface depends on the connectivity or integration of the cell membrane to other cellular structures such as actin, microfilaments, microtubules, and intermediate filaments (Ingber, 2008). The actin filaments associate with myosin filaments, and a mechanical tension is generated that can be distributed throughout the cell as well as to the external extracellular matrix (Ingber, 1993).

#### Cytoskeleton

The cytoskeleton is composed of three major types of protein filaments; microtubules, microfilaments and intermediate filaments. This scaffold can “sense” deformation at the cell surface and transmit it as a tension via focal adhesion sites, integrins, cellular junctions, and extracellular matrix (Davies, 2009). These tensional forces are transmitted to other parts of the cell or to the extracellular matrix and other cells.

#### Glycocalyx

Shear signaling is also initiated by the direct action of shear on structures on the apical membrane of the endothelium. One such structure is the endothelial glycocalyx, a fibrous net of carbohydrates that protrudes and covers the endothelial membrane. The glycocalyx is composed of heparan sulfate (HS), chondroitin sulfate (CS), and hyaluronic acid (HA). Mechanosensing by glycocalyx occurs via flow-induced tension acting on HS and CS. The biochemical signal triggered is then distributed by the cytoskeletal elements throughout the cell (Fu and Tarbell, 2013).

#### Integrins

Integrins are receptors that span across membranes and link cytoskeletal proteins with the endothelial matrix. Shear stress may be transferred by the cytoskeleton to integrins that distribute the force via actin microfilaments, microtubules, and intermediate filaments. This reorganization occurs via activation of focal adhesion kinase and c-Src kinases. The integrin αVβ3 has been reported to be flow sensitive and pretreatment with anti-αVβ3 antibody shows decreased shear induced signaling (Jalali et al., 2001).

#### Ion channels

One of the fastest endothelial responses to shear is activation of flow-sensitive ion channels. Some of these ion channels are candidate flow sensors. Inward rectifying K+ channel (Olesen et al., 1988) and outward rectifying Cl-channel (Barakat et al., 1999) have been reported to be activated by onset of flow. We reported on ATP-sensitive K+ channel (Chatterjee et al., 2006). We observed that this channel was responsive to removal of shear, i.e., it was deactivated by flow cessation. Other mechanosensitive channels ion channels are members of the TRP (Transient Receptor Potential) family such as TRPC1/TRPC3/TRPC6 and TRPV4. Mechanosensing TRPV4 channel facilitates the regulation of vascular growth in tumor ECs (Adapala et al., 2016). Besides conventional ion channels, another family of mechanosensitive ion channels are the Piezo channels, described in the following section.

#### Piezo

Piezo channels made up of Piezo proteins 1 and 2 are distinctly different from other mechanosensitive ion channels in that their structure is rather complex and their activation/inactivation kinetics are much more rapid than conventional ion channels. They also transduce very broad and varied kinds of mechanical inputs (Wu et al., 2017). Piezo 1 and 2 which act as mechanotransducers in mammalian cells are extremely large transmembrane proteins (35–40 transmembrane domains) that participate not merely in flow sensing but also in sensory perception, such as pain, touch, hearing etc. (Bagriantsev et al., 2014). Upon mechanical activation, Piezo currents are activated, but they desensitize rapidly as well. In ECs, Piezo1 currents have been reportedly to be activated with flow. In statically cultured ECs, Piezo are localized throughout the cell but under flow, they accumulate along the apical surface and seem to drive endothelial alignment in the direction of flow (Li et al., 2014).

#### Platelet-endothelial cell adhesion molecule (PECAM-1)

PECAM-1 is an adhesion molecule that is expressed along the cell-cell junctions of ECs. It is bound to several other structural moieties, viz integrin αvβ3, vascular endothelial growth factor receptor 2 (VEGFR2) (Tzima et al., 2005). Our work showed that PECAM-1 along with caveolin forms a mechanosensory complex (Noel et al., 2013). Elsewhere PECAM-1 has been reported to form a complex with the tyrosine-specific phospho-transferase Fyn (Chiu et al., 2008). A multimeric complex composed of PECAM-1, vascular endothelial growth factor receptor 2 (VEGFR2), and vascular endothelial cadherin (VE-cadherin) was sufficient to confer responsiveness to shear stress in cells (Tzima et al., 2005). PECAM-1 null mice showed an impaired response to reduction of shear stress in an *in vivo* model of reduced shear achieved by partial carotid artery ligation (Chen and Tzima, 2009; Chen et al., 2010). Isolated lungs from PECAM null mice showed a marked reduction in mechanosignaling (as evidenced by reduced ROS production) with stop of flow compared to wild type (Noel et al., 2013). These observations provide strong evidence for an important role of PECAM in the response of ECs to altered shear stress.

#### Caveolae

Caveolae, lipid rich invaginations on the endothelial cell surface, are reported to sense or transduce hemodynamic changes into biochemical signals that regulate vascular function. Caveolin-1, the major coat protein of caveolae, has been reported to show increased expression with increased shear and lack of caveolin-1 affects remodeling with altered blood flow (Yu et al., 2006). Our studies on stop of flow show markedly decreased mechanosignaling in ECs and intact lungs depleted of caveolae by genetic engineering (Noel et al., 2013). It is not clear how caveolae act as mechanosensors but it may be functionally linked to other mechanosensing molecules. We have shown recently that PECAM-1 is co-localized with caveolae as part of the mechanosensing machinery (Noel et al., 2013; Chatterjee and Fisher, 2014b).

#### The mechanosome

The mechanosome (a term that has been previously applied to stress signaling in bone) can be defined as a network of mechanosensors and transducers. Based on our earlier work, we posit that several mechanosensors on the endothelial cell membrane work as a mechanosome, i.e., caveolae, platelet endothelial cell adhesion molecule (PECAM), vascular endothelial growth factor receptor 2 (VEGFR2), vascular endothelial (VE)-cadherin and possibly other elements can sense and transduce shear signaling (Noel et al., 2013; Chatterjee and Fisher, 2014a).

## Transcriptional regulators of endothelial mechanotransduction

Shear stress activates several transcription factors (known as flow-regulated transcriptional factors) that in turn change the expression of a number of genes; the resultant changes in proteins affect endothelial responses, structure and function. Among flow regulated transcriptions factors are those of the Kruppel-like factor family, (KLF2 and KLF4), YAP (an effector of the Hippo signaling pathway), and NFκB (Hamik et al., 2007; Wang et al., 2016). Laminar blood flow which is well established to be atheroprotective and anti-inflammatory is found to induce the expression of KLF2 and KLF4 (Nayak et al., 2011). Once expressed, KLF2 causes increase in eNOS expression, and reduces endothelial permeability. KLF2 and 4 also reduce expression of inflammatory proteins on the endothelium. YAP is also regulated by the mechanical stimuli of shear stress; the resultant tension on the cytoskeletal components facilitates the translocation of YAP between the cytoplasm and nucleus. While unidirectional laminar flow causes nuclear translocation of YAP in a transient manner, disturbed or oscillatory flow facilitates nuclear translocation in a more sustained manner (Wang et al., 2016).

## Redox signaling in the vasculature (ROS, NO) with mechanotransduction

As we have described in an earlier section (Figure 1B), the first step in mechanotransduction is “sensing” of shear that occurs via the “mechanosensing machinery.” The next step is the transduction and transmission of this signal. Transduction has been reported by us and others (Chatterjee et al., 2012), to occur via kinases and other intermediates and leads to activation of a redox signal.

What are the redox signals generated by the endothelium with altered shear stress? Our work to date shows that stop of flow induced endothelial membrane depolarization is the trigger for activation of a PI3Kinase-Akt pathway. This pathway leads to the assembly of NADPH oxidase 2 and eventual ROS production (Figure 5). Elsewhere studies have shown that onset of flow induces endothelial membrane hyperpolarization that presumably causes ROS production, although the exact link has not been explored. We observed that depolarization of the endothelial membrane also led to activation of voltage gated calcium channels (VGCC). These channels that are expressed in ECs *in situ* (Gilbert et al., 2017) are lost when ECs are cultured for several passages. However, keeping cells under flow restored the channel expression (Wei et al., 2004). Our work shows that VGCC facilitates entry of calcium ions into the ECs leading to activation of endothelial nitric oxide synthase (eNOS) and subsequent NO production (Wei et al., 1999; Song et al., 2001; Figure 5). NO production has also reported with onset of flow (via transient increase of intracellular calcium; Davis et al., 2001; Sriram et al., 2016). Thus both ROS and NO is also released by the endothelium in response to both onset and cessation of shear stress (blood flow; McAllister and Frangos, 1999; Song et al., 2001).

The predominant source of ROS in the endothelium is NADPH oxidase (NOX). NOX has 7 isoforms (comprising of members NOX1-5 and Duox 1, 2) with NOX2 and 4 (and NOX1 primarily in smooth muscle cells underlying the endothelium) as the most abundant NOX in the vasculature (Pendyala et al., 2009). Work from several groups including ours has shown that NOX2 is localized in caveolar structures that “sense” shear as part of the mechanosome complex; this sensing facilitates NOX2 activation and ROS production (Rizzo et al., 2003; Yang and Rizzo, 2007; Noel et al., 2013).

ROS is a major signaling molecule that “transmits” the mechanosignal of shear stress into a biochemical response. ROS generated by the endothelium is in the form of superoxide (${\text{O}}_{2}^{-.}$) and, hydrogen peroxide (H_2_O_2_) which in turn gets converted to more potent oxidizing species such as hydroxyl radicals and hypochlorous acid (HOCl). The endothelium expresses both NADPH oxidase 2 (NOX2) and 4 (NOX4) which upon activation lead to the production of ${\text{O}}_{2}^{-.}$ and H_2_O_2_, respectively. ${\text{O}}_{2}^{-.}$ from NOX2 is produced in response to stop of shear (Chatterjee et al., 2010) but it is generated extracellularly (i.e., outside the endothelial cell membrane); it eventually dismutates to H_2_O_2_, but some of the superoxide directly enters the cells via anion channels (chloride channel 3) (Hawkins et al., 2007). This induces mitochondrial ${\text{O}}_{2}^{-.}$ generation which plays a role in activating the inflammasome pathway for onset and amplification of inflammation (Rimessi et al., 2016).

NOX4, that generates H_2_O_2_, has been reported to have a protective function. Indeed several studies have shown that mice lacking NOX4 had increased inflammation, hypertrophy of the medial layer in the blood vessels and endothelial dysfunction. Overexpression of NOX4 enhances vasodilation and reduces hypertension (Ray et al., 2011; Morawietz, 2018). This arises primarily from the link between NOX4 and hemeoxygenase-1 (HO-1); when NOX4 is disrupted the reduction in HO-1 leads to loss of activity of the transcription factor Nrf2 (as HO-1 regulates Nrf-2 activation; Schröder et al., 2012). Nrf2 is a well-established regulator of cellular protection against oxidative stress; it achieves this via upregulation of anti-oxidant enzymes. Thus, NOX4 induced Nrf2 activation is protective against endothelial dysfunction.

${\text{O}}_{2}^{\cdot -}$ (and its dismutated product) H_2_O_2_ also generates the highly reactive hydroxyl ion and radical (OH– and OH∙) via the Haber–Weiss reaction. Both OH– and OH∙ are very strong oxidizing species and cause oxidative damage to membrane proteins and lipids. In the presence of polymorphonuclear neutrophils (PMN), H_2_O_2_, and chloride generate HOCl, a process that is catalyzed by the PMN specific enzyme myeloperoxidase (MPO). MPO reportedly acts as an NO oxidase. The resultant reduction in NO, increases oxidative stress and exacerbates endothelial dysfunction (Korthuis, 2018).

Besides ROS, NO is the other important signaling molecule that participates in “transmission” of the mechanical signal associated with blood flow. In this, the caveolar structures play a pivotal role. NO is produced primarily via activation of the endothelial nitric oxide synthase (eNOS) that localizes in caveolar structures on the cell membrane. eNOS is activated when it dissociates from caveolin-1. This is followed by its phosphorylation at different serine residues and association with calmodulin (a calcium activating protein). Laminar flow activates eNOS by its dissociation from caveolin and phosphorylation (serine 635 and serine1179) (Fulton et al., 1999). eNOS transcription and expression have also been reported to be laminar shear dependent; indeed a transient increase in eNOS mRNA has been reported with onset of flow although this was found to stabilize with long term exposure to shear stress (Davis et al., 2001; Ishibazawa et al., 2011). Low or turbulent shear stress lead to decrease in eNOS phosphorylation and thus reduction in NO in the vasculature (Malek et al., 1999).

ROS and NO are the transmission signals in the mechanotransduction cascade. Indeed, ROS production is linked to NO generation as ROS can act as a sink for NO resulting in the production of peroxynitrite (ONOO^−.^) that causes endothelial damage, dysfunction and the production of pro-inflammatory cytokines, chemokines, and growth factors. Thus elevated levels of ROS lead to low NO bioavailability, as has been reported in ECs exposed to irregular flow. Reduced bioavailability of NO creates a pro-oxidant milieu resulting in increased oxidative stress. NO regulates the vascular environment by inhibiting proinflammatory cytokines, cell adhesion molecules etc. and by facilitating vasodilation to improve blood flow.

ROS produced by the endothelium leads to activation of transcription factors NFκB, AP-1, and hypoxia inducing factor 1α (HIF-1α), as we reported in our studies using “cessation of shear” models (Figure 5). These transcription factors regulate several growth and cell differentiation proteins. Among these are VEGF which was reported by us and others to be upregulated by altered shear (Browning et al., 2014). In our model of stop of blood flow achieved by ligation of the femoral artery, ROS was found to induce HIF-1α activation within minutes of stop of flow prior to hypoxia as monitored by reduction of pO2 values. We found that ROS induced increase in VEGF and HIF-1α are responsible for endothelial growth that drives revascularization with stop of flow (Browning et al., 2014).

Overall the endothelium responds to onset of shear in a manner similar to cessation of shear stress; i.e., the signaling pathways are similar and are activated by a change in shear.

### Mechanosensing and the regulation of vascular inflammatory signaling

Vascular inflammation necessitates the activation of the endothelium so as to facilitate interaction between the circulating immune cells and the endothelium. The endothelial activation, as we have mentioned earlier, is in the form of increased expression of CAMs and selectins, and in the release of pro-inflammatory cytokines interleukin-4 (IL-4) and 13 (IL-13) and chemokines tumor necrosis factor α (TNFα), IL-1 (Davies, 1993, 2008; Davies et al., 1995). These are signals that lead to recruitment and adherence of leukocytes, PMN and other immune cells to the site of injury; indeed close proximity of immune cells to the endothelium is known to be followed by rolling and adhesion to the endothelial layer aided by adhesion molecules, cytokines etc.

Regions of turbulent flow in the vascular tree show upregulation in the expression of CAMs such as ICAM-1 and VCAM-1 in the endothelium. The integrin-CAM complex increases leukocyte-endothelium adhesion and extravasation into the vascular layer, a key step in development of atherosclerosis. Turbulent shear thus plays is a major role on the development of atherosclerosis at certain locations of the vascular tree.

Under certain conditions, ROS can drive onset of inflammation via upregulation of some inflammatory moieties such as intercellular adhesion molecule (ICAM-1), vascular cell adhesion molecule (VCAM-1), and damage associated molecular patterns (DAMPs) as we have reported in the past (Orndorff et al., 2014; Jungraithmayr et al., 2016; Tao et al., 2016). DAMPs are a class of ligands that are expressed and released in response to a sterile injury presumably via ROS production. Once expressed DAMPs are released into the circulation where they can bind to their respective receptors on the surface of ECs. In the next section, the ROS-inflammation link in the context of endothelial mechanotransduction will be discussed in detail.

### Mechanotransduction and vascular disease

Forces associated with shear are a major determinant for regulation of vessel diameter. As mentioned in earlier sections, atherosclerosis, or the formation of plaques and fibrous caps on vessel walls occurs in those regions of the vascular tree where flow patterns are disturbed. Work over the past decade has shown that endothelial mechanotransduction plays a major role in activating atheroprotective or atherogenic genes based on blood flow patterns.

Under physiological conditions, the endothelial cell-cell barrier is well regulated and vascular permeability is tightly controlled by complex junctional structures, namely adherens junctions (AJ), tight junctions (TJ), and gap junctions (GJ) (Radeva and Waschke, 2017). Under various stimuli these junctions change structurally to regulate permeability. With pathological conditions introduced due to various risk factors as well as in regions of turbulent flow, pro-inflammatory signals activate CAMs and alter AJs (Melchior and Frangos, 2010). Long term shear-induced reorganization of the actin cytoskeleton, to which AJs are linked, can also influence the distribution of these junctions and consequently lead to increased permeability. Indeed regions of turbulent flow show a destabilized endothelial barrier which facilitates penetration of leukocytes, T-lymphocytes, and monocytes/macrophages into the endothelial layer and the arterial intima. Thus increased permeability contributes to the pathogenesis of atherosclerotic vascular disease (Daniel and van Buul, 2013).

Junctional structures (AJ, TJ, GJ) as well as junctional proteins such as occludin and VE-cadherin have also been suggested as potential mechanotransducers that sense the blood flow and contribute to the conversion of mechanical forces to the intracellular signaling (Hahn and Shchwartz, 2009). Low shear has been reported to downregulate expression of junctional proteins such as occludin and to promote its phosphorylation (Conklin et al., 2007) which lead to decreased vascular integrity (DeMaio et al., 2001).

ROS and NO produced as a result of mechanotransduction also play a role in regulating endothelial TJ and permeability. ROS induced inflammatory signals drive PMN recruitment; PMN and inflammatory mediators in turn act on the endothelial junctional proteins and TJs to alter barrier function. ECs exposed to ROS have been reported to show an increased permeability that is directly linked to disruption of TJs due to altered phosphorylation state of junctional adhesion molecules (Rodrigues and Granger, 2015).

ROS induced reduction in endothelial barrier function has also been attributed to an alteration in NO bioavailability. However, NO has been reported as both a negative and a positive modulator of endothelial barrier function (Kubes and Granger, 1992). NO can be protective by limiting the PMN-endothelial cell adhesion. Negative modulation is presumably via NO induced S-nitrosylation of eNOS which inhibits its activity. Inhibition of eNOS increases the permeability of endothelial cell monolayers via formation of stress fibers and disruption of junctional proteins (Durán et al., 2010).

### Mechanotransduction and revascularization

*In vivo* studies of aberrant flow showed vascular remodeling in terms of thickening of the blood vessels or in terms of driving an “inflammatory” phenotype. In contrast to aberrant shear, our studies with stop of flow models revealed increased proliferation of ECs (Browning et al., 2014) which was abrogated by blocking shear induced signaling either by inhibiting depolarization or by blocking ROS production. We also discovered that cell proliferation was linked to angiogenesis and revascularization. This revascularization that rescued the impeded blood flow was abolished in mice that were administered with agents to block mechanosignaling i.e. to block depolarization or ROS production (NOX2 assembly) and in mice with null of K_ATP_ channel or NOX2 genes. Conditional knockouts (endothelial NOX2 expressors on a null background) showed revascularization indicating that endothelial NOX2 was crucial to the mechanosignaling response (Browning et al., 2014). We thus concluded that endothelial mechanosignaling with stop of flow was an attempt by the vasculature to restore impeded flow via angiogenesis and revascularization (Browning et al., 2014).

### Understanding mechanotransduction with I/R: implications in lung transplant

The transplant scenario, associated with stop and restart of blood flow represents an altered mechanotransduction paradigm. In most systemic organs, transplant would cause (1) alteration in shear stress and (2) altered oxygen delivery to the organ. This “hypoxia-reoxygenation” signaling overshadows the signaling associated with altered mechanotransduction. Hypoxia-reoxygenation is well established to lead to activation of the xanthine-xanthine oxidase pathway and also the mitochondrial respiratory chain and NADPH oxidase pathway. All of these events result in the formation of oxidants and eventual tissue injury.

Unlike systemic organs, the lung does not obtain its oxygen supply from blood flow; rather alveolar oxygen can keep lung cells “oxygenated” even in the absence of blood flow. Similarly reperfusion of the lung does not increase oxygen supply. Thus pulmonary I/R can be considered to represent a mechanosignaling or mechanotransduction paradigm. Our studies on lung I/R demonstrated that stop and restart of flow caused the production of ROS and led to tissue injury. Since the lung does not become hypoxic with altered blood flow, we investigated the mechanism by which ROS is produced.

**Figure 6:**
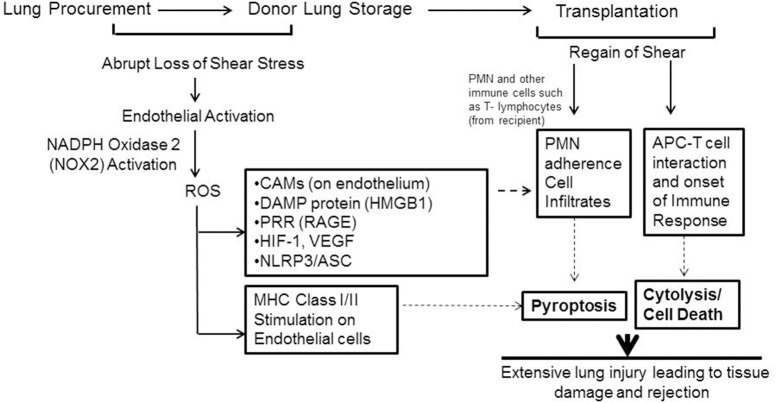
Schema showing mechanotransduction induced signaling as would occur with organ storage. ROS activates an inflammation cascade during storage. These predispose the graft to bind to immune cells that influx into it after transplant. ROS can also transform the lung endothelial cells as an antigen presenting cell (APC). The endothelium as APC can “present” donor antigens to the T-lymphocytes that come from the recipient. The APC-T cell interaction leads to cytokine release and cytolysis. (Originally published in Jungraithmayr et al., 2016, permission to reproduce obtained from publisher).

What is the fate or effect of the ROS produced in the lungs (or grafts) during ischemia (storage)? Our investigations using murine and human lungs showed that ROS led to production of moieties that triggered the onset of an inflammatory cascade (Jungraithmayr et al., 2016; Tao et al., 2016). Stored or ischemic lungs showed high expression of ICAM-1 and VCAM-1. As we have mentioned earlier, both these moieties facilitate adherence of PMN, macrophages, monocytes etc. to the endothelial layer (Figure 6). Additionally the DAMP protein high mobility group box protein (HMGB1) and its receptor RAGE were also upregulated as a function of storage time (Tao et al., 2016). Increase in HMGB1 and are RAGE critical in onset and amplification of inflammation and have been implicated in numerous inflammation pathologies (Wang et al., 1999; Kokkola et al., 2005; Lamkanfi et al., 2010). Indeed the HMGB1–RAGE axis triggers inflammatory cascades by activation of multimeric complexes or inflammasomes (Lamkanfi and Kanneganti, 2010).

Human donor lungs are usually stored under two-thirds inflated conditions prior to transplant and thus continue to be oxygenated; however, if HIF-1α (and thus VEGFA) induction occurred under normoxic conditions of lung storage this would initiate proinflammatory signaling in donor lungs, even in the absence of hypoxia. We also reported on the induction of the HIF1α-VEGF axis in stored or ischemic lungs (Tao et al., 2016) which also participates in driving influx, recruitment and adherence of PMN and other immune cells (Figure 6). Furthermore, ROS generated by the endothelium during storage can trigger an adaptive immune response in the form of transformation of lung endothelium as an antigen presenting cell (APC). APCs participate in adaptive immune response by “presenting” donor antigens to the T-lymphocytes from the recipient that enter into the graft after transplant. The activated endothelium of the graft can thus facilitate T cell recruitment and activation; these are well established to lead to cytokine release and cytolysis. The cumulative damage from these events causes injury and onset of rejection post-transplant (Jungraithmayr et al., 2016).

Thus mechanosignaling during lung storage and transplant needs to be minimized in order to reduce graft injury. Toward that end, new strategies advocating for continued perfusion of donor lungs have been adopted. Indeed perfusion of donor lungs is now being increasing employed to (1) prevent damage during storage, (2) preserve the graft over long periods of time, and (3) repair grafts that have been partially damaged so that they may be used for transplantation. We propose that blocking the mechanosignaling cascade by introducing the “perfusion-ventilation” maneuver or by the maintaining of physiological conditions during storage, would maintain graft health. Studies with animal lungs indicate that a relatively low perfusate flow rate can prevent activation of the “loss of shear stress” signaling cascade. Of note, maximal increase in shear signaling occurs with the transition of perfusate flow from 1.8 ml/min to zero (Al-Mehdi et al., 1998).

## Shear signaling and therapeutics

Our current knowledge on shear stress induced signaling and its role in endothelial integrity and/or in endothelial dysfunction can potentially inform various clinical strategies to protect against shear stress induced endothelial damage. One of the crucial players in shear signaling is ROS; thus delivery of phytopharmaceuticals that can bolster the antioxidant defense can facilitate endothelial protection. For instance, low or abnormal shear induced oxidative stress in ECs can be reduced by the antioxidant resveratrol (Wang et al., 2014). Additionally, the use of agents that drive anti-inflammatory genes or proteins may be employed to reduce endothelial injury arising from abnormal or disturbed shear. Agents such as tannic acid that can increase KLF2 expression on the endothelium can be used to treat atherosclerotic vascular disease (Xu et al., 2017). The protection conferred on the endothelium by KLF2 is well established and thus other agents that function as KLF2 activators have also been suggested for beneficial cardiovascular effects. Resveratrol (which reportedly induces KLF2 expression) and cholesterol lowering statins (which regulate KLF2 expression) drive an atheroprotective phenotype in the endothelium (Parmar et al., 2005).

## Conclusions and future perspectives

Significant advances have been made in recent years toward understanding the various subcellular players and sequence of molecular events that are associated with endothelial mechanotransduction specifically in the context of vascular health and disease. However, the major challenges remain in understanding how mechanosignals integrate at the tissue and organ level across complex cellular environments to generate a biological response. It is also important to analyze and evaluate the collective interaction between mechanosignaling and chemical signals (risk factors etc.) that play a pivotal role in vascular responses to shear.

## Author contributions

The author confirms being the sole contributor of this work and approved it for publication.

### Conflict of interest statement

The author declares that the research was conducted in the absence of any commercial or financial relationships that could be construed as a potential conflict of interest.
